# Increased Temporal Dynamics of Intrinsic Brain Activity in Sensory and Perceptual Network of Schizophrenia

**DOI:** 10.3389/fpsyt.2019.00484

**Published:** 2019-07-12

**Authors:** Youxue Zhang, Gang Guo, Yuan Tian

**Affiliations:** ^1^School of Psychology, Chengdu Normal University, Chengdu, China; ^2^School of Foreign Languages, Chengdu Normal University, Chengdu, China

**Keywords:** schizophrenia, functional connectivity, temporal variability, self-disorder, sensory and perceptual network

## Abstract

Schizophrenic subject is thought as a self-disorder patient related with abnormal brain functional network. It has been hypothesized that self-disorder is associated with the deficient functional integration of multisensory body signals in schizophrenic subjects. To further verify this assumption, 53 chronic schizophrenic subjects and 67 healthy subjects were included in this study and underwent resting-state functional magnetic resonance imaging. The data-driven methods, whole-brain temporal variability of fractional amplitude of low-frequency fluctuations and regional homogeneity (ReHo), were used to investigate dynamic local functional connectivity and dynamic local functional activity changes in schizophrenic subjects. Patients with schizophrenia exhibited increased temporal variability ReHo and fractional amplitude of low-frequency fluctuations across time windows within sensory and perception network (such as occipital gyrus, precentral and postcentral gyri, superior temporal gyrus, and thalamus). Critically, the increased dynamic ReHo of thalamus is significantly correlated with positive and total symptom of schizophrenic subjects. Our findings revealed that deficit in sensory and perception functional networks might contribute to neural physiopathology of self-disorder in schizophrenic subjects.

## Introduction

About 1% of the whole adult population suffer from schizophrenia, which is one of the costliest mental disorders. Schizophrenic subject is typically considered as a self-disorder (1). Self-disorder could be associated with several positive symptoms. The major point of schizophrenic subjects’ positive symptom is unable to efficiently distinguish self and others. This symptom would lead to a worse deficit that the schizophrenic patients could not confirm their actions and thoughts are related to external information or stimulation. Importantly, in schizophrenia, the symptoms related to self-disorder have been considered a crucial factor to identify whether the psychiatric patient is schizophrenic or not (2).

There are many neuroimaging studies that have been employed in investigating the neuropathological mechanism of schizophrenia (3–5). Although many functional connectivity studies of schizophrenia focused on the abnormal long-range functional connectivity among spatially distributed brain regions (6, 7), few studies paid attention on local functional information of blood oxygen level dependence and functional interaction between spatially adjacent regions (8, 9). Thus, to quantify local or short-range functional connectivity in human brain, several measures were commonly employed in neuroimaging studies, including regional homogeneity (ReHo) (10), local power of blood oxygen level dependence [low-frequency fluctuations (fALFF)] (11), and functional connectivity strength (12) derived from resting-state functional magnetic resonance imaging (fMRI). Several studies have reported that there are significant relationships between static ReHo/fALFF and several factors, such as age, gender, and intelligence in healthy subjects (13, 14). These findings have revealed that the static local neural activity and short-range functional connectivity have been linked with the physiological and psychological factors in human brain.

In schizophrenia, multi-site resting-state fMRI study has shown that schizophrenic subjects exhibited decreased static fALFF in cuneus, middle temporal gyrus, and posterior cingulate cortex compared with healthy subjects (15). Guo et al. has also found that the schizophrenic patients showed both decreased static fALFF in the posterior cingulate cortex and decreased gray matter volume in medial prefrontal cortex, indicating that the changes of brain function and anatomy within default model network might contribute separately to the pathophysiology of schizophrenia (16). Besides, recent studies have indicated that schizophrenic patients have shown reduced static functional connectivity density in primary sensory network of schizophrenia and decreased static ReHo in visual and sensorimotor networks compared with healthy controls (17). Furthermore, the symptomatology (e.g., auditory hallucinations) in schizophrenia has been proved to be related to abnormal multisensory static functional connectivity (18). In conclusion, the deficit static functional connectivity of sensory and perceptual systems may potentially contribute to physiopathology of schizophrenia. While these studies have implicitly revealed that functional connectivity is a stable characteristic across the entire resting scan period, recent studies have indicated that functional connectivity is not stationary and changes over time (19, 20).

Assessing brain dynamic functional connectivity from resting-state fMRI has advanced our knowledge of the brain (21). Specifically, a recent neuroimaging study has stated that functional connectivity variability seems to be a reliable feature, partly dependent on functional relationships among distributed brain regions (22). Dynamic functional connectivity analysis could provide a novel method to sensitively capture the abnormal functional connectivity related with psychiatric disorders (23–26). The results of dynamic functional connectivity analyses also revealed transient states of dysconnectivity in schizophrenia (27, 28), which support and expand current knowledge regarding dysconnectivity in schizophrenia. Moreover, a recent study demonstrated that the feature of dynamic functional connectivity significantly outperforms the static connectivity in classification analysis (29). These findings reveal that static functional analysis may obscure important dynamic features of network behavior.

During recent years, few studies have focused on altered local temporal variability of functional activity or short-range functional connectivity in schizophrenia, which could reveal information that is not from static functional connectivity (30). Thus, we sought to determine whether altered temporal variability of regional neural activity was associated with symptom of schizophrenia in this study. The dynamic neural activity analysis used in this study includes dynamic ReHo and fALFF, which allow us to identify voxel-level dynamic functional alterations in schizophrenia compared with healthy subjects. On the basis of previous results about abnormal static functional connectivity in primary motor and perception networks, we hypothesize that abnormal dynamic neural activity in schizophrenia would locate in primary perceptual systems, such as primary sensory-motor cortex and related visual and thalamus regions. In addition, schizophrenic subjects are expected to show significant association between altered variability of these network and symptom of schizophrenic subjects.

## Materials and Methods

### Subjects Selection and Schizophrenic Patients’ Clinical Symptoms

Fifty-three chronic schizophrenic subjects and 67 healthy controls are included in this study. Related resting-state fMRI data are collected from the Center for Biomedical Research Excellence. The patients with schizophrenia are diagnosed according to Diagnostic and Statistical Manual of Mental Disorders, 4th Edition, diagnostic. The psychiatric symptom severity is measured using positive and negative syndrome scale (PANSS) assessment. Healthy subjects are also recruited, those who do not have schizophrenia and not exhibiting Axis I symptoms. These research procedures were in accordance with institutional review boards of the USA. Written informed consent was obtained from each subject before the study. Details of demographic characteristics of both groups are shown in **Table 1**.

**Table 1:** Dataset (The Center for Biomedical Research Excellence, chronic).

	Patients with Schizophrenia	Healthy controls	*p*
Sample size	53	67	–
Gender (Male/Female)	42/11	46/21	0.192^a^
Age (years)	36.75 ± 13.67	34.82 ± 11.28	0.398^b^
Education level (years)	13.20 ± 1.82	14.02 ± 1.86	0.024^b^
Handedness (both/right/left)	1/42/10	1/65/1	0.004^a^
FD	0.15 ± 0.07	0.14 ± 0.08	0.433^b^
Disease duration (years)	14.94 ± 4.60	–	–
PANSS-positive score	14.94 ± 4.61	–	–
PANSS-negative score	14.43 ± 5.26	–	–
PANSS-global score	30.07 ± 8.28	–	–

Indicated values are shown as mean ± standard deviation. PANSS, positive and negative symptom scale; FD, Framewise displacement.

a Indicates the p values from the comparison analysis (Chi-square test).

b Indicates the p values from the comparison analysis (two-sample t-test).

### Data Acquisition and Image Preprocessing

Functional imaging scan was performed on a 3T MRI scanner (Siemens Trio). Resting-state functional image are collected with single-shot full k-space echo-planar imaging (EPI) (repetition time = 2,000 ms, echo time = 29 ms, number of slices = 32, slice thickness = 3 mm, matrix size: 64 × 64, flip angle = 7°, field of view = 256 × 256 mm^2^). Subjects underwent 6-min scan. A total of 180 volumes of EPI images were obtained.

The preprocessing steps of functional image were performed using commonly processing steps [Data Processing and Analysis of Brain Imaging (DPABI) (31), http://rfmri.org/dpabi] and briefly described here. First, temporal and spatial corrections were performed, including slice time and head motion correction, furthermore normalized (voxel size: 3 mm) into EPI template. Any subjects who had a maximum translation in any of the cardinal directions larger than 3 mm or a maximum rotation larger than 3° were excluded from subsequence analysis. Moreover, framewise displacement (FD) was evaluated in two groups as suggested by Power et al. (32). Second, detrending analysis was performed on the normalized data to minimize the effect of linear trend. Third, several nuisance signals were regressed out from functional image through linear regression analysis. The nuisance signals include six motion parameters and their first temporal derivative, white matter and cerebrospinal fluid signals. In this study, the global signal was not removed from the functional image (33, 34).

### Temporal Variability Analysis

**Figure 1:**
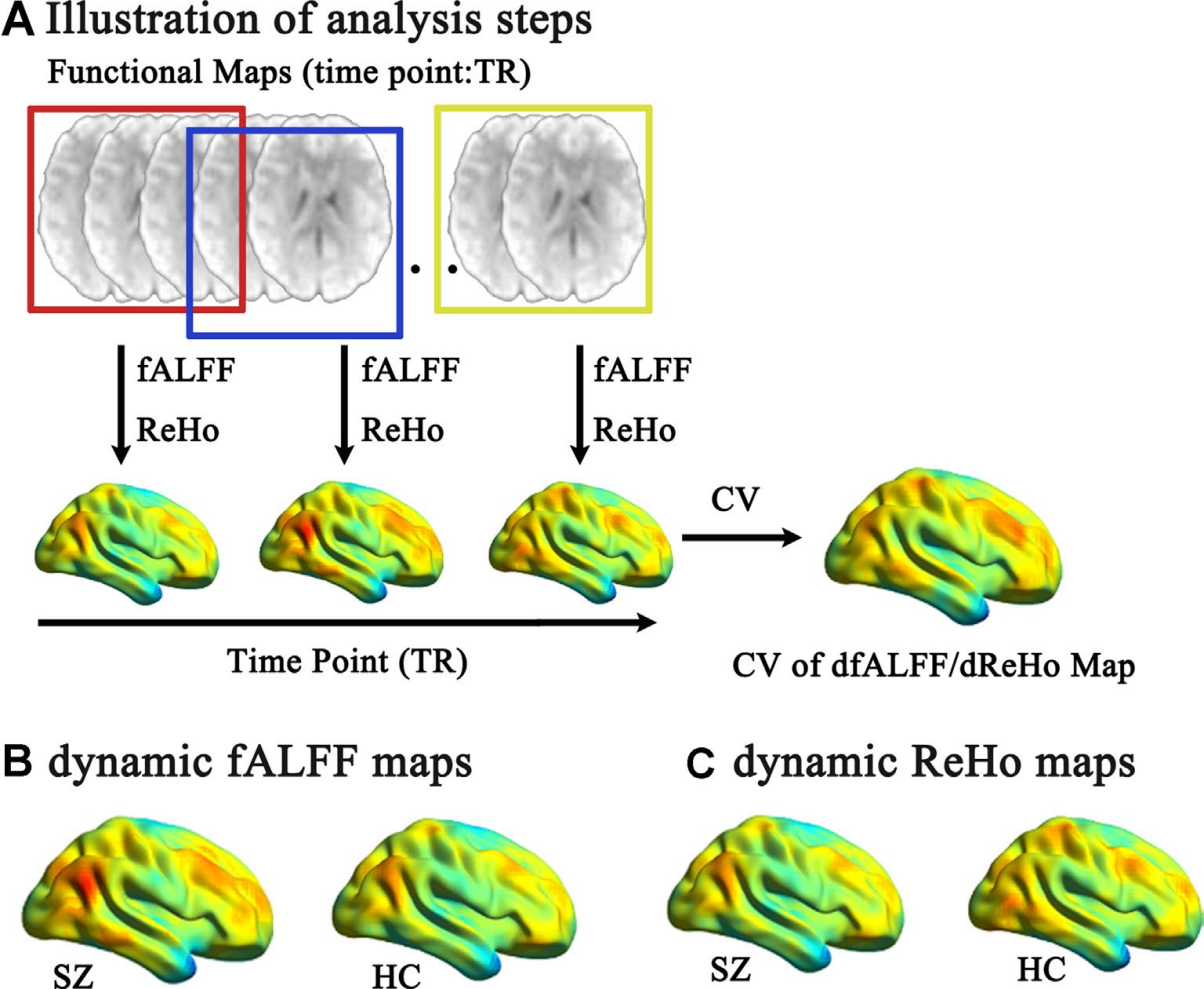
Illustration of analysis steps and temporal variability of dynamic fALFF and ReHo pattern. **(A)** The preprocessed full-length blood oxygen level-dependent fMRI maps were segmented into several sliding windows (50 TR). Within each window, the fALFF and ReHo were computed for each voxel. The sliding window was systematically shifted by one TR, and the corresponding fALFF and ReHo were computed. Then, the temporal variability of the dynamic fALFF and ReHo were defined as the CV maps across the sliding windows. The pattern of temporal variability of the fALFF **(B)** and ReHo **(C)** of the schizophrenic subjects/healthy controls were shown.

Two widely used approaches, including ReHo and fALFF, were used to measure voxel-level functional maps (35). We calculated dynamic ReHo and fALFF through sliding window analysis (**Figure 1A**). Based on the “rule of thumb,” which is 1/f_min_ of data should be equal or less than the length of window (36), the whole-run time series of each voxel was segmented into 50 TR windows and sliding the onset of these windows by one TR. Then, within each window, we calculated ReHo and fALFF at each voxel in whole-brain mask.

In the ReHo analysis, the frequency band passing (0.01–0.08 Hz) was done on fMRI data. Then, Kendall’s W value was calculated for each voxel, between the time series of the target voxels and the series of their nearest voxels (26 voxels) in the whole-brain mask (10). In the fALFF analysis, fALFF is defined as the percentage of the power within the low-frequency range (0.01–0.08 Hz) in total power of whole frequency range (0–0.25 Hz) (11).

Across *n* window, we calculated the coefficient of variation (CV) maps of ReHo and fALFF for each subject. We define the CV of a voxel *k* as:

$$CV_{k}=\frac{\sqrt{\sum_{t=1}^{n}{\left(x_{t}-x_{\mathrm{mean}}\right)}^{2}/n}}{x_{\mathrm{mean}}}$$

where *x* *_t_* is ReHo or fALFF score of voxel *k* over time window *t*, *t* = 1,2,…,*n*; *x* *_mean_* is mean score of *x* *_t_* across time window *t*. Finally, individual voxel-wise ReHo and fALFF CV maps were standardized by dividing the whole-brain mean values and, furthermore, spatially smoothed (6-mm full width at half maximum of the Gaussian kernel). Then, two-sample t-tests were performed for ReHo and fALFF CV maps, respectively (DPABI, http://rfmri.org/dpabi), between schizophrenic and healthy subjects with age, gender, education level, handedness, and FD as covariates, with a statistical significance level corrected by false discovery rate (*p* < 0.05).

### Correlations With Pathological Factors

We assessed the association between the score of clinical score and significant changes of temporal variability in regional functional measurements in patients with schizophrenia. We extract the mean CV score from the peak voxel and its nearest voxels (26 voxels) for each significant cluster. Then, the partial correlation analysis was performed between ReHo and fALFF CV scores and patients’ PANSS scores with age, gender, education level, handedness, medication dosage, and FD as covariates (*p* < 0.05).

### Validation Analysis

Recent fMRI study has indicated that sliding window-based dynamic functional connectivity could be largely explained by head motion (37). Patient is chronic schizophrenic subjects in this study. The antipsychotic treatment might have an effect on dynamic local neural activity of schizophrenic subjects. Thus, we preformed the validation analysis to investigate the influence of these factors on dynamic temporal variability of regional functional measurements in schizophrenic subjects.

First, spike-regression-based scrubbing was performed to take into account transient head motion (38, 39). We defined the “bad points” with high FD (above 0.5 mm) and their adjacent time points (1 back and 2 forward) for each subject. These “bad points” were modeled as separate regressor in the nuisance regression models in the preprocessing analysis. Then, for new preprocessed fMRI data, we reevaluated the temporal variability of ReHo and fALFF through sliding window analysis. Two-sample t-tests were also performed between two groups with age, gender, education level, handedness, and FD as covariates. Second, to take account of antipsychotic treatment, we calculated the relationship between altered temporal variability of fALFF/ReHo and medication dosage in schizophrenia group by using correlation analysis (*p* < 0.05).

## Results

### Temporal Variability of fALFF/ReHo Between Schizophrenic and Healthy Groups

**Figure 2:**
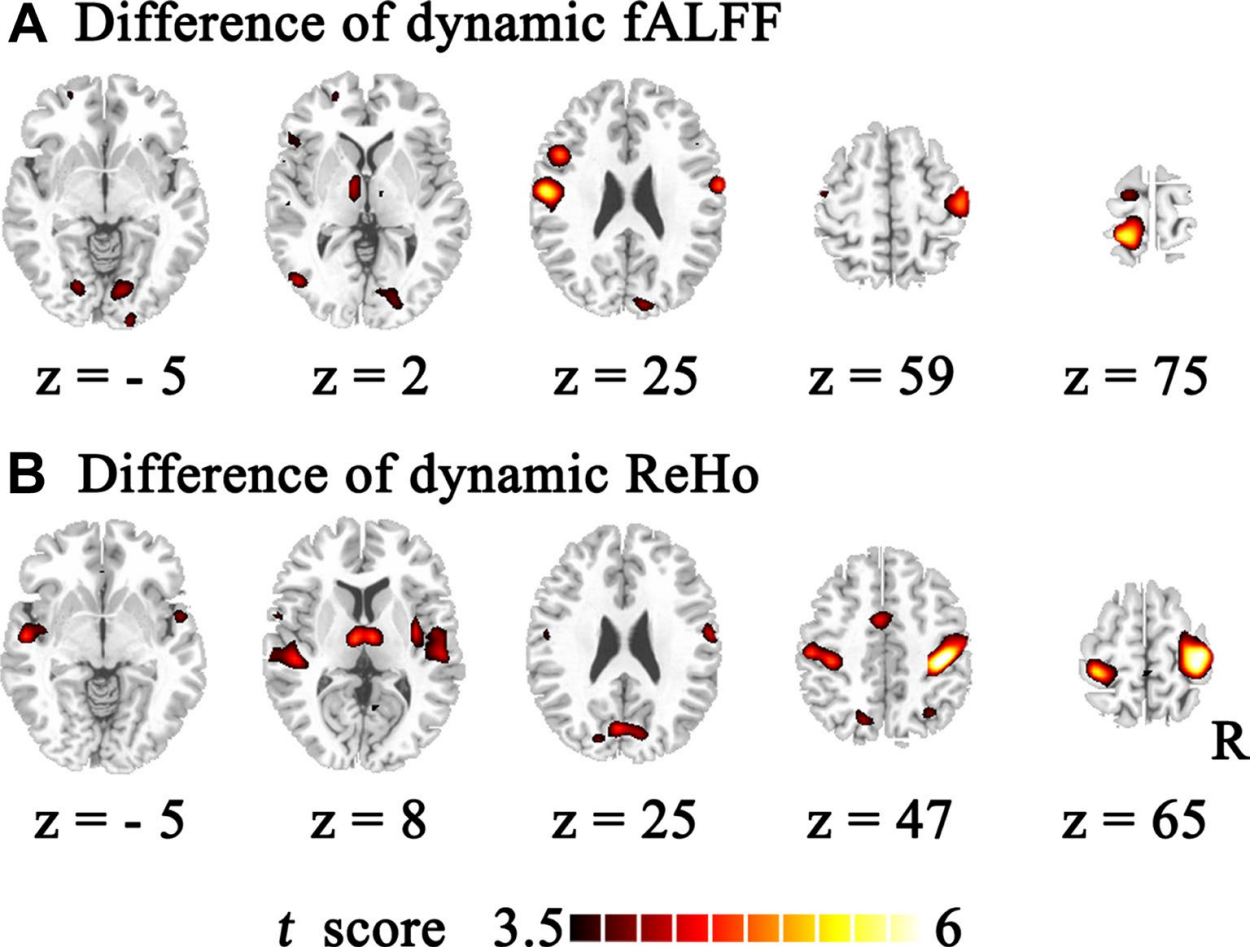
Group difference of temporal variability of the dynamic fALFF and ReHo. Temporal variability of the dynamic fALFF and ReHo between schizophrenic and healthy subjects were identified using two-sample *t* tests. The significance level was set *P* *_FDR_* < 0.05. **(A)** The increased dynamic fALFF in schizophrenic subjects were compared with those of healthy controls. **(B)** The enhanced dynamic ReHo in patients with schizophrenia.

Temporal variability of dynamic fALFF and ReHo were shown at each voxel for each subject (**Figures 1B, C**) with the BrainNet viewer (http://www.nitrc.org/projects/bnv/) (40). The variability of these dynamic local neural activity displayed a nonuniform spatial distribution across the brain. The lowest variability was located in the limbic system. The largest variability was mainly located in the heteromodal association region, including the temporal–parietal junction, prefrontal and posteromedial cortex. The primary sensory and visual cortices showed a moderate level of variability. Furthermore, using two-sample *t* test, schizophrenic subjects showed increased temporal variability in both dynamic fALFF and ReHo compared with healthy controls (**Table 2**, **Figure 2**) with the DPABI viewer (41). Within temporal variability of fALFF, increased dynamic fALFF were observed in thalamus, super temporal gyrus, precentral/postcentral gyrus, and lingual gyrus in schizophrenic subjects. Similar increased dynamic ReHo were also being found in patients with schizophrenia, including super temporal gyrus, thalamus, postcentral gyrus, middle cingulum cortex, and cuneus. Furthermore, these findings were observed by using spike-regression-based scrubbing procedure (**SFigure 1**).

**Table 2:** Significant increased dynamic fALFF and ReHo in schizophrenic subjects.

Regions	MNI coordinates			Peak t-score	Cluster voxels
	x	y	z		
Dynamic fALFF					
Left postcentral gyrus	−57	−12	21	5.992	763
Left precentral gyrus					
Left superior temporal gyrus					
Right postcentral gyrus	48	−21	60	5.097	452
Right precentral gyrus					
Left postcentral gyrus	−18	−42	75	5.844	228
Left precuneus					
Left superior parietal gyrus					
Right lingual gyrus	9	−81	−9	3.921	60
Left Thalamus	−9	−12	0	3.970	33
Dynamic ReHo					
Left postcentral gyrus	−30	−39	66	5.549	1,363
Left superior parietal gyrus					
Right cuneus					
Left cuneus					
Left precentral gyrus					
Left superior temporal gyrus					
Left temporal gyrus					
Right postcentral gyrus	39	−30	48	6.401	1,326
Right precentral gyrus					
Right superior temporal gyrus					
Right rolandic operculum					
Right insula					
Right heschl gyrus					
Left Middle temporal gyrus					
Middle cingulum cortex	−6	0	42	4.727	121
Supplementary motor area					
Left thalamus	−6	−12	6	4.899	108
Right thalamus					

### Correlations With Pathological Factors

**Figure 3:**
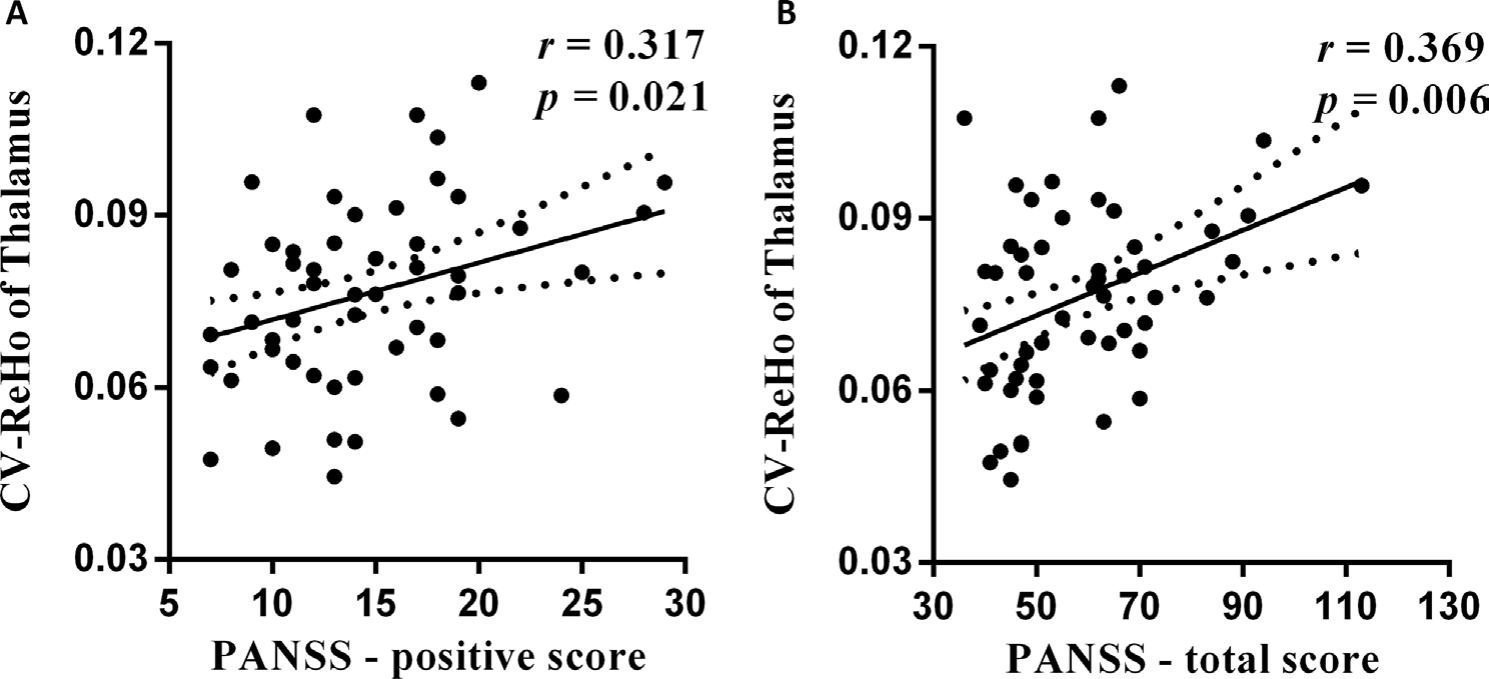
The relationship between altered temporal variability of dynamic ReHo and PANSS scores. **(A)** The positive association is observed between increased CV score of thalamus region and PANSS-positive score in schizophrenic subjects. **(B)** The PANSS-total score was also positively related with CV score of thalamus region in patients.

We observed positive correlation between PANSS scores and the increased temporal variability of ReHo in schizophrenic subjects: PANSS-positive score and thalamus within basal ganglia network (BGN) (*r* = 0.317, *p* = 0.021, **Figure 3A**) and PANSS-total score and thalamus within BGN (*r* = 0.369, *p* = 0.006, **Figure 3B**). The relationship was observed between PANSS-total score and thalamus within BGN by using spike-regression-based scrubbing procedure (**SFigure 2**). Moreover, no other significant correlations were found between the altered temporal variability of fALFF/ReHo and medication dosage in schizophrenia group.

## Discussion

This study has presented some new insights in alterations of dynamic temporal variability of ReHo and fALFF in schizophrenia through sliding window analysis. Consistent with our hypothesis, increased dynamic temporal variability of ReHo and fALFF were observed in sensory and perceptual networks in schizophrenic subjects. Critically, the psychiatric symptom analysis has indicated that increased temporal variability of ReHo showed significantly positive relationship with the positive symptoms of schizophrenic subjects. These findings provide evidence that there is deficient temporal variability of local neural activity in low-level perceptual processing in schizophrenic subjects.

While these are well known about the abnormal higher-order brain function in schizophrenia, such as memory and cognitive (42, 43), neuroimaging studies have also documented some basic sensory processing deficits in schizophrenic subjects. The perceptual deficits have been increasingly observed in the sensory networks, including primary motor and visual regions (44, 45). A recent study has also revealed that schizophrenic subjects has shown increased resting-state functional connectivity variability in sensory and perceptual networks (46). Most of these locations were in line with the meta-analysis’ results of schizophrenia (47). Increased variability of local neural activity of sensorimotor regions might reflect the deficits in the integration of multisensory stimuli in schizophrenia (48). Moreover, enhanced dynamic local neural activity might indicate that the abnormal bottom–up processing is associated with the pathological mechanism of schizophrenia (46). In this study, we observed increased temporal variability of ReHo and fALFF in sensory and perceptual system across time windows in schizophrenic subjects. These increased local temporal variabilities might provide some new evidences to support deficient dynamic neural activity in primary sensorimotor, as well as the abnormal dynamic bottom–up processing in schizophrenia.

Furthermore, schizophrenic patients could be commonly considered as a self-disorder with abnormal functional network (49). Recent studies have revealed that the processing and integration of multisensory bodily signals underlay a coherent self-experience in healthy controls (50, 51). In the “rubber-hand illusion” experiment, Botvinick and Cohen pointed out that the subjects would have true self-experience when they saw the fake hand was stroked, synchronous individual’s unseen hand (50). Disturbances in self-experiences were also reported by Ehrsson; they found that visual perception was not match with proprioceptive information (51). These studies have provided the evidence that the sense of self-experiences depend on multisensory information that arose from the body, such as proprioceptive, spatial, and temporal sensorimotor signals. In schizophrenia, the deficits of visual and motor networks appear to be related to self-disorder (46, 52, 53). Besides, the neurobiological model of self-disorder has also indicated that deficient sense of self in schizophrenia is largely related to the abnormal multisensory signals integration from body and external stimuli (54, 55). Thalamus is a very crucial key role in gating and in integrating multisensory and cognitive information in human brain. Thus, previous studies have indicated that the altered static function of the thalamus is an important feature related to the schizophrenic subjects’ self-disorder symptom (56, 57). In this study, we found increased temporal variability of ReHo and fALFF in primary visual and somatosensory area in schizophrenic patients. These increased dynamic neural activity across time may be related to the high interaction within regional sensorimotor functional network in schizophrenia. Increased temporal variability of thalamus was also observed in schizophrenic subjects, which may suggest that abnormal dynamic functional integration across time in schizophrenia exists between multisensory regions and higher order cognitive functional system. A significant relationship was observed between increased dynamic ReHo of thalamus and PANSS-positive score. These findings indicated that schizophrenic subjects have altered dynamic local functional connectivity and local dynamic neural activity in thalamus regions. Moreover, increased local dynamic functional connectivity of the thalamus maybe related with a positive symptom of schizophrenic subjects. Therefore, the abnormal dynamic local neural activity within the visual, sensorimotor, and thalamus areas might provide more evidence about abnormal self-processing in schizophrenia.

While our results provide a new insight of dynamic functional activity for understanding the self-disorder in schizophrenia, several main methodological points of this study should be further addressed. First, dynamic temporal variability of ReHo and fALFF were calculated through sliding window correlation analysis. The size of window length is one parameter that does not have formal consensus, although we selected it based on the frequency of preprocessed data. Second, the patient we chose is chronic schizophrenic subjects. The antipsychotic treatment might have an effect on dynamic local neural activity of patients. We should validate our findings in the first-episode schizophrenic subjects in further study. Third, self-experience assessment is not included in the current study. We should measure it and investigate the relationship between self-experience score and static/dynamic local neural activity in schizophrenic subjects.

## Conclusion

In conclusion, this study has combined resting-state fMRI and dynamic functional analysis. Our findings have revealed an increased temporal variability of ReHo and fALFF in primary visual and sensorimotor networks, as well as in the thalamus in schizophrenia patients. It has been showed that the increased dynamic neural activity of the thalamus was significantly related with a positive symptom of schizophrenic subjects. Thus, our findings might have potential interpretation for the neural physiopathology of self-disorder in schizophrenia.

## Author Contributions

YZ made a substantial contribution to the conception and drafting and revising of the article. YZ, GG, and YT made a substantial contribution to the analysis and interpretation of the data, and gave final approval of the version to be published.

## Funding

This scientific work was supported by grants from the National Nature Science Foundation of China (grant number: 81801775) and Advanced Talents Introduction Program of Chengdu Normal University (YJRC2017-4).

## Conflict of Interest Statement

The authors declare that the research was conducted in the absence of any commercial or financial relationships that could be construed as a potential conflict of interest.
