# Supplementary material for: Increased Temporal Dynamics of Intrinsic Brain Activity in Sensory and Perceptual Network of Schizophrenia
Source: Front Psychiatry. 2019 Jul 12;10:484. doi: 10.3389/fpsyt.2019.00484 (PMC6639429; doi:10.3389/fpsyt.2019.00484)
Supplement: Supplementary file 1 [file DataSheet_1.pdf]

Supplementary material

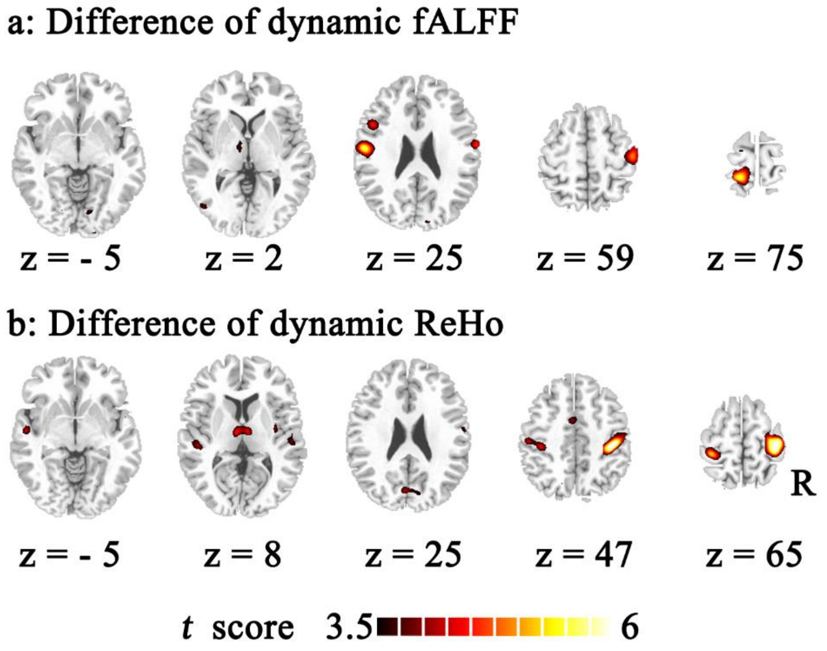

SFigure 1: Group difference of temporal variability of the dynamic fALFF and ReHo. Temporal variability of the dynamic fALFF and ReHo between schizophrenic and healthy subjects were identified using two-sample *t* tests. The significance level was set $P_{FDR} < 0.05$. *(a)* the increased dynamic fALFF in schizophrenic subjects compared to healthy controls. *(b)* the enhanced dynamic ReHo in patients with schizophrenia.

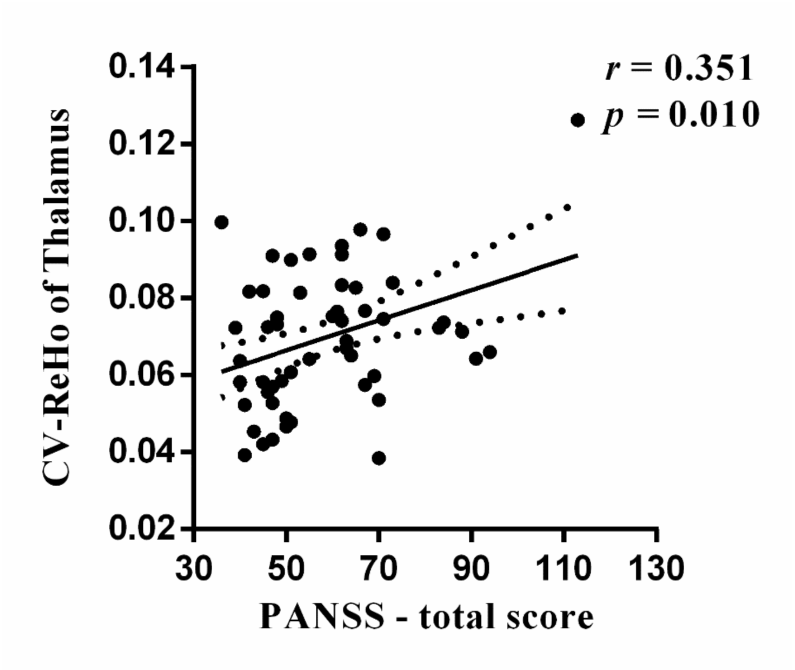

SFigure 2: The PANSS-total score was positively related with CV score of thalamus region in schizophrenic subjects.
